# Phylodynamic and Evolution of the Hemagglutinin (HA) and Neuraminidase (NA) Genes of Influenza A(H1N1) pdm09 Viruses Circulating in the 2009 and 2023 Seasons in Italy

**DOI:** 10.3390/pathogens13040334

**Published:** 2024-04-17

**Authors:** Fabio Scarpa, Leonardo Sernicola, Stefania Farcomeni, Alessandra Ciccozzi, Daria Sanna, Marco Casu, Marco Vitale, Alessia Cicenia, Marta Giovanetti, Chiara Romano, Francesco Branda, Massimo Ciccozzi, Alessandra Borsetti

**Affiliations:** 1 Department of Biomedical Sciences, University of Sassari, 07100 Sassari, Italy; aciccozzi@uniss.it (A.C.); darsanna@uniss.it (D.S.); 2 National HIV/AIDS Research Center (CNAIDS), Istituto Superiore di Sanità, 00162 Rome, Italy; leonardo.sernicola@iss.it (L.S.); stefania.farcomeni@iss.it (S.F.); 3 Department of Veterinary Medicine, University of Sassari, 07100 Sassari, Italy; marcasu@uniss.it; 4 Laboratorio di Biologia Molecolare—Fondazione Università Niccolò Cusano, 00166 Rome, Italy; marcovitale0409@gmail.com (M.V.); alessiacicenia@virgilio.it (A.C.); 5 Department of Sciences and Technologies for Sustainable Development and One Health, Universita Campus Bio-Medico di Roma, 00128 Rome, Italy; giovanetti.marta@gmail.com; 6 Instituto René Rachou, Fundação Oswaldo Cruz, Belo Horizonte 30190-009, MG, Brazil; 7 Climate Amplified Diseases and Epidemics (CLIMADE), Brasilia 70070-130, DF, Brazil; 8 Unit of Medical Statistics and Molecular Epidemiology, University Campus Bio-Medico of Rome, 00128 Rome, Italy; chiara.romano@unicampus.it (C.R.); f.branda@unicampus.it (F.B.); m.ciccozzi@unicampus.it (M.C.)

**Keywords:** influenza A virus, 2009 H1N1, pandemic, evolution, Italy

## Abstract

The influenza A(H1N1) pdm09 virus, which emerged in 2009, has been circulating seasonally since then. In this study, we conducted a comprehensive genome-based investigation to gain a detailed understanding of the genetic and evolutionary characteristics of the hemagglutinin (HA) and neuraminidase (NA) surface proteins of A/H1N1pdm09 strains circulating in Italy over a fourteen-year period from 2009 to 2023 in relation to global strains. Phylogenetic analysis revealed rapid transmission and diversification of viral variants during the early pandemic that clustered in clade 6B.1. In contrast, limited genetic diversity was observed during the 2023 season, probably due to the genetic drift, which provides the virus with a constant adaptability to the host; furthermore, all isolates were split into two main groups representing two clades, i.e., 6B.1A.5a.2a and its descendant 6B.1A.5a.2a.1. The HA gene showed a faster rate of evolution compared to the NA gene. Using FUBAR, we identified positively selected sites 41 and 177 for HA and 248, 286, and 455 for NA in 2009, as well as sites 22, 123, and 513 for HA and 339 for NA in 2023, all of which may be important sites related to the host immune response. Changes in glycosylation acquisition/loss at prominent sites, i.e., 177 in HA and 248 in NA, should be considered as a predictive tool for early warning signs of emerging pandemics, and for vaccine and drug development.

## 1. Introduction

Influenza A viruses belonging to the Orthomyxoviridae family are segmented single-strand-negative sense RNA viruses that infect a variety of hosts, including wild birds, humans, and pigs [1]. Genetic changes at critical positions on the surface glycoproteins haemagglutinin (HA) and neuraminidase (NA), either by amino acid substitutions (antigenic drift) or by segment swapping (antigenic shift), can lead to an efficiently transmitted emerging virus to which humans are not immune, and which can cause a pandemic [1]. In March 2009, the pandemic influenza A virus (H1N1) pdm09, commonly referred to as “swine flu”, first emerged in Mexico and then spread rapidly worldwide through human-to-human transmission, triggering the first influenza pandemic of the 21st century [2]. In June 2009, the World Health Organization (WHO) issued a phase 6 pandemic alert, officially declaring the start of the 2009 influenza pandemic [3]. In Italy, only limited transmission occurred in autumn/winter 2009, characterized by a wave that peaked in mid-November and subsided in December 2009, while a second epidemic wave was recorded between November 2010 and March 2011, in which the A(H1N1) pdm09 virus was responsible for most of the infections [4]. The 2009 pandemic H1N1 virus (H1N1pdm09) contains a unique combination of gene segments from human, swine, and avian influenza A viruses derived from the reassortment of North American H3N2 and H1N2 swine viruses (triple-reassorting swine viruses (PB2, PB1, PA, HA, NP, and NS genes) and Eurasian swine H1N1 viruses (M and NA genes) [1,2]. Transmission of 2009 A(H1N1) pdm09 from humans to pigs has occurred repeatedly in recent years, and has increased genetic diversity in pigs [5,6,7,8], while cases of swine-to-human transmission are less frequent [5,6,7,8,9]. Cross-species transmission events such as human-to-swine-to-human transmission of A(H1N1) pdm09 to swine workers could lead to unpredictable outcomes and epidemiologic consequences. In addition, the high mutation rate of the influenza A virus due to the error-prone RNA-dependent RNA polymerase leads to the production of many antigenically distinct viral variants, resulting in high genetic diversity and an accumulation of amino acid changes in the surface glycoproteins HA and NA, allowing the virus to escape immunity induced by vaccination or previous infection [1,2]. Virologic surveillance of influenza is critical for identifying changes in viral fitness that pose a potential pandemic threat, and for predicting the emergence of circulating influenza viruses for seasonal vaccine formulations. The H1N1pdm09 virus has displaced previously circulating seasonal H1N1 viruses (e.g., Bris/59) and continues to circulate and evolve [10]. Phylogenetic analysis of the hemagglutinin (HA) gene of A(H1N1) pdm09 viruses has revealed at least seven major clades distributed worldwide [11,12,13,14,15]. In this study, we performed a genetic and phylodynamic investigation of the HA and NA sequences of A(H1N1) pdm09 Italian isolates to determine the evolutionary trend of the human pandemic influenza virus circulating in Italy between 2009 and 2023.

## 2. Materials and Methods

In order to accomplish the predetermined objectives, five datasets were assembled using all available sequences from the GISAID database (https://gisaid.org Accessed Date 1 March 2024). The initial dataset was constructed to illustrate the variability of Italian lineages over a broad temporal scale, encompassing all Italian HA sequences from 2009 to 2023 (n = 1623). Subsequent datasets focused on Italian HA and NA sequences isolated in 2009 and 2023 (HA_09 = 306, NA_09 = 188, HA_23 = 136, NA_23 = 69). Each dataset underwent independent alignment using the L-INS-I algorithm implemented in Mafft 7.471 [16]. Manual editing was conducted using Unipro UGENE v.35 [16]. The software jModeltest 2.1.1 [16] was utilized to identify the most suitable probabilistic model of genome evolution through a maximum likelihood optimized search. To depict Italian lineage variability over a broad temporal scale, the dataset comprising all HA sequences (from 2009 to 2023) was dated using Bayesian Inference (BI) conducted with BEAST 1.10.4 [17], involving runs of 200 million generations under various demographic and clock models. Model selection for the most representative output was determined through the test of Bayes Factor [17] by comparing 2lnBF values of marginal likelihoods, by means of the software Tracer 1.7 [18]. The reliability of the dataset was verified by conducting phylogenetic signal verification using likelihood mapping analyses [18] and the software Tree-Puzzle [14], analyzing 10,000 random quartets. The software BEAST was further utilized to investigate the phylogenetic relationships among terminals in the four datasets (HA_2009, NA_2009, HA_2023, NA_2023), with runs of 200 million generations under various demographic and clock models, and model selection followed the same procedure as previously described. The time-calibrated tree was not constructed for these four datasets due to the absence of a temporal signal. 

Principal Coordinate Analysis (PCoA) was performed using GenAlEx 6.5 [19] to identify genetic structuring, potential subgroups within genetic clusters, and to assess genetic variability among isolates. The aim of this analysis was to evaluate the dissimilarity represented by the genetic variability among the analyzed isolates. The PCoA reconstruction was based on a pairwise p-distance matrix of genetic data estimated with 1000 iterations, using the R package APE [20]. Runs were finalized by using the option PCoA via a covariance matrix with data standardization. To identify sites under selection, the FUBAR (Fast, Unconstrained Bayesian AppRoximation) [21] method was implemented in the Datamonkey platform [22] and has been applied to the following four datasets: HA_2009, NA_2009, HA_2023, and NA_2023. Runs were performed under a Bayesian-based approach for detecting pervasive selection. This analysis allows us to provide a detailed understanding of the evolutionary dynamics of the present sequences.

## 3. Results

### 3.1. Phylogenetic Reconstruction of Global and Italian A(H1N1) pdm09 Epidemics 

**Figure 1:**
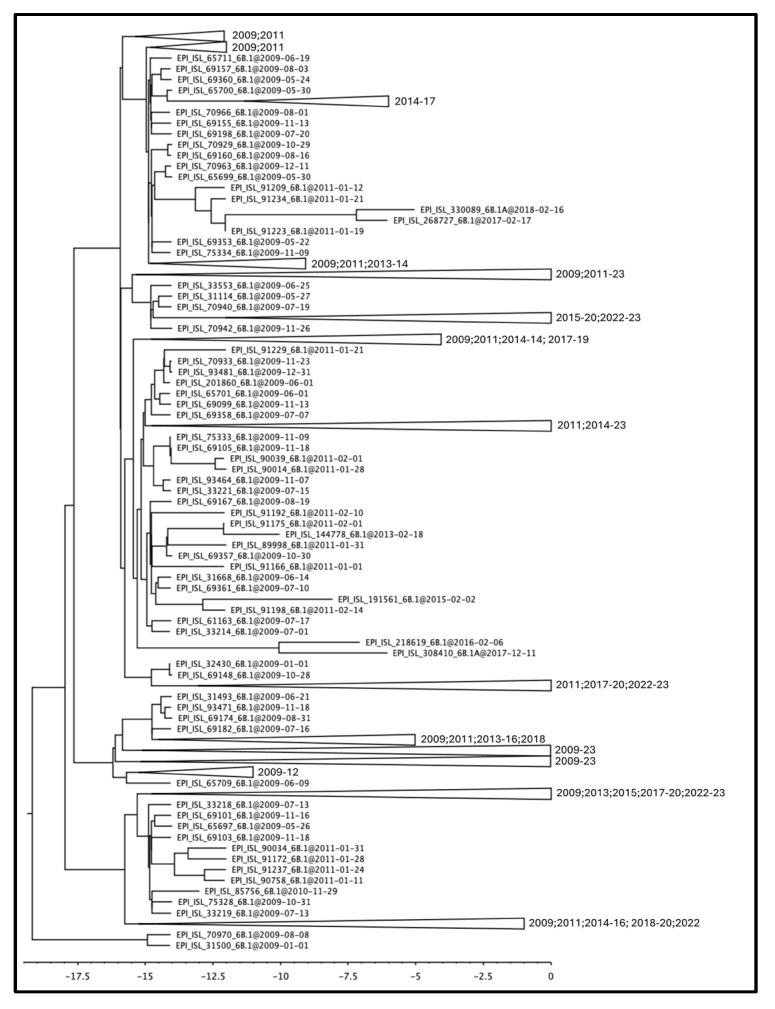
Time-scaled phylogenetic tree of the Italian A(H1N1) pdm09 HA sequences sampled between 2009 and 2023. All nodes are highly supported (posterior probabilities > 0.95). The scale below is expressed in years before 2023. Terminals constituting genetically monophyletic groups have been collapsed, and the labels on the right indicate the sampling years. Figure S1 represents the tree in its fully extended form.

The obtained time-scaled phylogenetic tree containing the HA gene of the A(H1N1) pdm09 Italian isolates belonging to the H1N1 strain from 2009 to 2023 shows a complete absence of genetic structuring over the temporal scale (Figure 1). The tree does not reveal the presence of any clade representing a coherent discrete group depending on the date of sampling. In the tree, it can be observed that the older lineages from the 2009 to 2011 pandemic period are positioned at the basal level, suggesting that the subsequent bifurcations that have occurred derived from them (see Figure S1 for details on single terminals). The branches containing the more recent terminal nodes are separated by a greater distance.

### 3.2. Genetic Variations of A(H1N1) pdm09 Virus Isolates from 2009 and 2023

**Figure 2:**
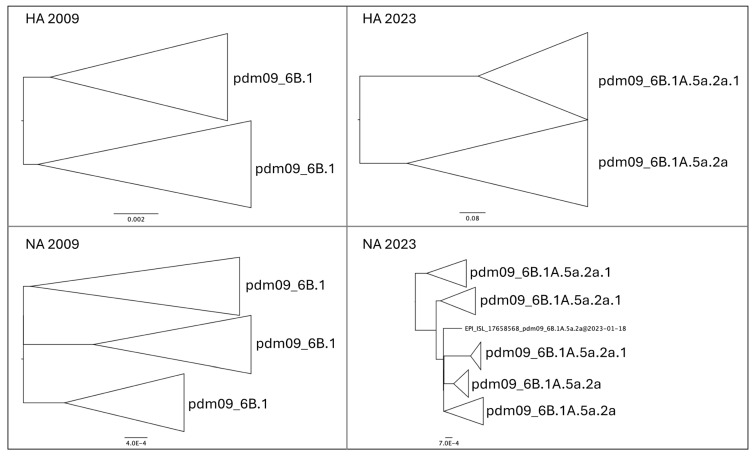
Phylogenetic reconstruction of Italian isolates of influenza A H1N1pdm09 HA and NA gene sequences circulating in Italy in 2009 and 2023.

The phylogenetic analyses of the HA gene of the H1N1pdm09 lineage exhibited a different time-based temporal structure (Figure 2). Samples isolated in 2009 showed the occurrence of two main groups belonging to clade 6.B1, with few genetic differences, but without forming new clades or subgroups. The phylogeny depicts short internal branches interspersed mainly at the root of the tree, indicating rapid spread, and increasing in genetic diversity during the early phase of the pandemic, with several dead-end transmissions (see Figures S2 and S3). In contrast, isolates from 2023 changed their evolutionary pattern, resulting in a typical feature of human seasonal influenza viruses. The isolates from 2023 were split into two main groups representative of two clades, i.e., 6B.1A.5a.2a and its descendant 6B.1A.5a.2a.1. The NA sequences from isolates of 2009 were clustered into three main groups, with few genetic differences and without forming new clades or subgroups (see Figures S4 and S5). The NA sequences from isolates of 2023 were split into five groups with the two clades of 6B.1A.5a.2a and its descendant 6B.1A.5a.2a.1 dispersed without constituting monophyletic groups.

**Figure 3:**
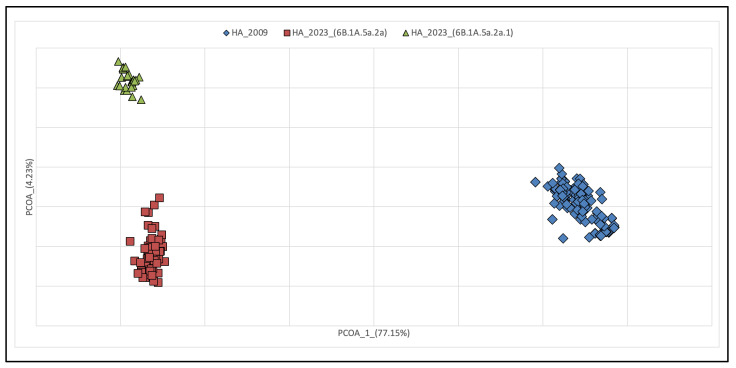
Principal Coordinates Analysis of HA in 2009 and 2023 with groups set a priori. Symbols indicate each sequence depicted as a data point in 2009 and 2023. Bidimensional plot shows the genetic differentiation among sequences due to the nucleotide substitutions per site found in the dataset. The cumulative variability explained by the first three axes amounts to 83.82% (Axe 1: 77.15; Axe 2: 4.23; Axe 3: 2.44).

**Figure 4:**
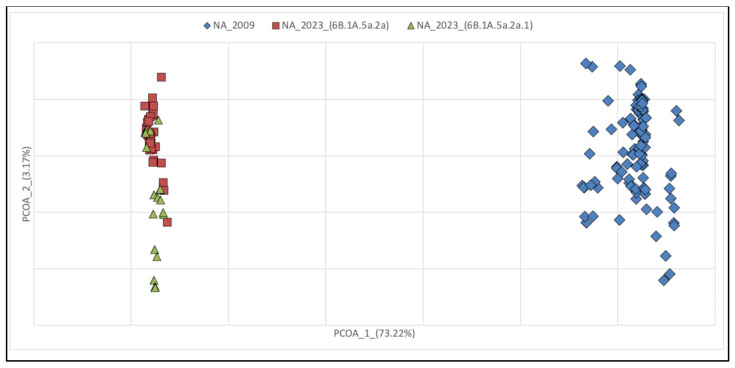
Principal Coordinates Analysis of NA in 2009 and 2023 with groups set a priori. Symbols indicate each sequence depicted as a data point in 2009 and 2023. Bidimensional plot shows the genetic differentiation among sequences due to the nucleotide substitutions per site found in the dataset. The cumulative variability explained by the first three axes amounts to 79.09% (Axe 1: 73.22; Axe 2: 3.17; Axe 3: 2.71).

Overall, phylogenetic analyses revealed a different evolutionary pattern of NA and HA genes, with greater diversification observed within HA than in NA genes in 2023 compared to 2009. These results were also confirmed by analyses of principal coordinates (PCoA) (Figure 3 and Figure 4). PCoA based on genetic data is a powerful tool to evaluate the viral population diversity within and between lineages. In the HA gene PCoA, the isolates from 2009 and 2023 are separated by the first axis. However, the isolates from 2023 are further subdivided along the second axis, with two groups representing clades 6B.1A.5a.2a and 6B.1A.5a.2a.1. In the NA gene PCoA, the isolates from 2009 and 2023 are separated, but in the latter, they were not further subdivided based on their clades of origin. 

**Figure 5:**
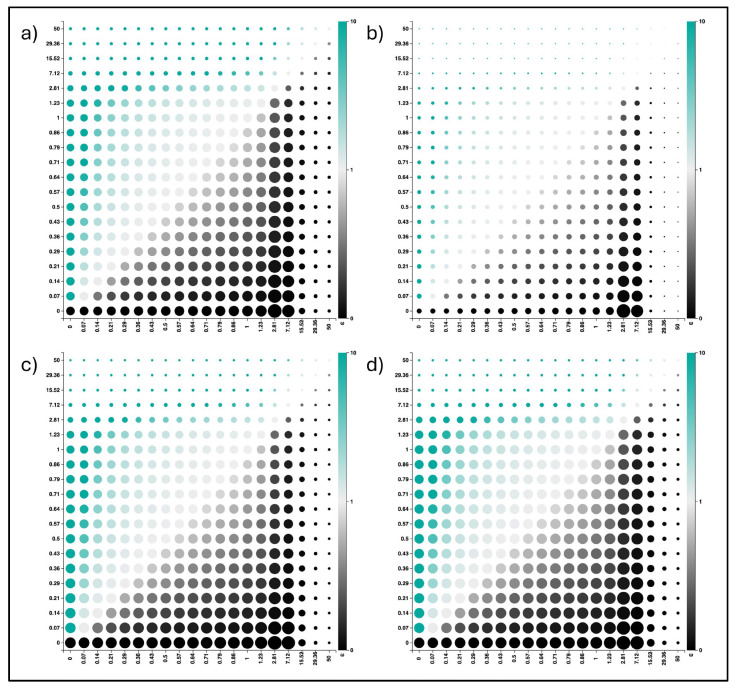
(**a**) HA 2009; (**b**) HA 2023; (**c**) NA 2009; (**d**) NA 2023. Graph showing the posterior rate distribution across the discretized rate grid of the ω parameter, which assesses natural selection within molecular sequences (indicated by the colored bar on the right of each graph). ω > 1: positive selection; ω = 1: neutrality; and ω < 1: negative selection. X axis indicates synonymous substitution rate. Y axis indicates non-synonymous substitution rate. The dot’s size corresponds to the posterior weight assigned to each grid point, while the color indicates the strength of selection. Sites under positive selection are highlighted in green, while those under negative selection are highlighted in black.

The simulation carried out using the FUBAR method identified a total of nine sites with positive selection in the various datasets; two of them were in the HA 2009 dataset, three were in the HA 2023 dataset, three were in the NA 2009 dataset, and one was in the NA 2023 dataset (Table 1). In addition, the simulation identified fifty-six sites under negative selection, including five in the HA 2009 dataset, twenty-seven in the HA 2023 dataset, seventeen in the NA 2009 dataset, and seven in the NA 2023 dataset. Figure 5 shows the posterior distribution over the entire alignment of synonymous and non-synonymous sites, revealing that the number of sites under negative selection for the HA gene increased over time, while it decreased for the NA gene. The estimated mean on each individual dataset of the parameter α/β, which identifies the ratio between synonymous and non-synonymous mutations, amounts to 3.33, 3.74, 3.36, and 3.43 for HA_09, HA_23, NA_09, and NA_23, respectively.

## 4. Discussion

The antigenic variability of the influenza A virus poses a major global threat as it can cause periodic catastrophic pandemics [23]. Therefore, it is important to maintain epidemiological and virological surveillance, with vaccination remaining the best prevention strategy. Here, we described the genetic variability and the evolutionary characteristics of the HA and NA genes of A(H1N1) pdm09 influenza viruses in Italy, with a focus on the earliest viruses isolated at the beginning of the 2009/2010 pandemic compared to those collected during the 2023 season. To this end, we performed an analysis that included all accessible genomes deposited in the GISAID database from 2009 to 2023.

Genomic analysis of virus sequence data from Italy in 2009 revealed the introduction of clade 6B.1 in Italy, with few genetic differences between the isolates, while in the 2023 season, most isolates fell into clade 6B.1A.5a.2a and its descendant 6B.1A.5a.2a.1, without forming new clades or subgroups.

Overall, the phylogenetic reconstruction reveals the lack of genetic structuring. In particular, the phylogeny of the HA molecule in the period between 2009 and 2023 shows no temporal structuring, with all terminals falling into clades present in the tree without forming discrete groups. It is noteworthy that all terminals representing pandemic isolates (2009–2010–2011) are positioned basally relative to the others, showing the ancestral variability from which all other lineages originated. The length of the branches carrying older isolates is much shorter than the more recent ones, indicating a slowdown in the evolutionary rate over the years. This is entirely normal for a virus that no longer needs to adapt to the host. In fact, the beginning of the epidemic was characterized by a rapid increase in genetic diversity, in the absence of strong selective pressure following the invasion of a naïve host population. The high mutation rate is the biological characteristic required for adaptation to the new host, and this is a condition that repeats itself proportionally regardless of the evolutionary potential of the virus, as was also the case at the onset of the COVID-19 pandemic. This condition remains constant regardless of the geographical scale, both globally and regionally. The rate of evolution then tends to slow down when the virus becomes more “endemic” in the host population, which typically reflects a lower virulence and dangerousness of the virus. The HA gene seems to present a faster rate of evolution, as can be seen from both the phylogenetic tree and the PCoA. Indeed, HA not only forms groups that separate 2009 and 2023 isolates, but also splits isolates belonging to the two 2023 clades of (H1N1)pdm09. In contrast, NA shows no segregation in 2023 and forms polyphyletic and paraphyletic groups in the tree. This is also confirmed by the dispersion of points in the PCoA of the NA fragment, where individuals are widely scattered over the diagram area, indicating not only a lack of structuring but also variability that is obviously not subject to selection. 

It is interesting to note that in the context of antigenic drift and antigenic shift, if the molecular variability of HA exceeds that of NA, this may indicate greater antigenic changes in HA compared to NA. Antigenic drift refers to gradual, small changes in the antigen over time due to the accumulation of point mutations [24]. Higher variability in HA indicates increased mutational pressure over time, leading to greater genetic and antigenic diversity in HA than in NA. Antigenic shift involves sudden and significant changes in viral antigens, often resulting from genetic recombination between human and animal influenza viruses [24]. If HA shows greater variation than NA, this could indicate that significant structural changes mainly affect HA, making it a more likely target for antigenic shift phenomena. In either case, higher genetic variability in HA may affect the ability of the influenza virus to evade pre-existing immunity, and has important implications for diagnosis, epidemiologic surveillance, and influenza vaccine development.

Moreover, the test for site under selection pressure corroborates this observation. Indeed, the simulation showed that the number of sites under negative selection increased over time for the HA gene, while it decreased for the NA gene. The observation of an increase in sites undergoing negative selection and a decrease in sites subject to positive selection during the evolution of a virus over time provides valuable insights into the adaptive strategies and evolutionary dynamics of the virus. An increase in sites under negative selection may indicate that the virus is evolving into a less virulent form. This could be due to selection pressure that favors variants that are less detrimental to host survival [25]. Conversely, the decrease in sites under positive selection suggests that the virus has reached an equilibrium with its host. This could indicate the development of strategies to evade the host immune system or an effective host immune response against previously positively selected sites [25]. Additionally, the increase in sites under negative selection might indicate an evolution towards greater genetic stability, influenced by reduced selection pressure or decreased genetic diversity within the virus population. 

Overall, the parameter indicating the ratio between synonymous and non-synonymous mutations is always higher than one, and has similar values. This suggests that synonymous mutations are more frequent than non-synonymous mutations. From an evolutionary perspective, this could indicate a more conservative selection pressure or relative stability in protein sequences, where non-synonymous mutations are less tolerated compared to synonymous mutations. The dynamic balance between different evolutionary factors could provide a broader interpretation of a consistent ratio of alpha to beta that goes beyond the mere difference in the distribution of sites under positive and negative selective pressure. This equilibrium may suggest that, despite variations in selection pressure on specific sites, the gene is subject to a range of evolutionary influences that maintain an alpha to beta ratio that is stable over time. Indeed, the negative selection pressure, which could arise from deleterious mutations, might be counterbalanced by factors such as genetic drift or recombination, contributing to the overall genetic diversity and the stability of protein sequences [26]. This dynamic balance between the different evolutionary processes could be crucial for the maintenance of protein function and structure throughout the evolutionary history of the gene. It is important to consider variations in the evolutionary history of the virus, such as random selection or genetic bottleneck events, which could also affect the distribution of sites under positive and negative selection over time [27]. 

N-linked glycosylation (NLG) of HA and NA proteins plays an important role in the antigenicity, transmission, and pathogenicity of the virus [28,29]. The seasonal influenza H1N1 viruses prior to 2009 had more glycosylation sites on the HA than the 1918 and 2009 pandemic viruses. The absence of glycosylation at residue 177 of the HA after the 2009 pandemic suggests possible changes in the immunogenic features of the protein. Glycosylation at residue 177 of HA was only observed in a few strains isolated from Thailand (2009–2012) and China (2011) [30]. The addition of this site to the A(H1N1) pdm09 virus has been associated with resistance to neutralizing antibodies in vitro [31]. The N248D substitution in NA, which has been correlated with increased viral stability that became fixed at a global level, may contribute to the adaptation of the virus to the human immune system [32].

Our analysis confirmed a previous study [12] that also found evidence for positive selection of sites 177 in HA and 248 in NA in the 2009 datasets, suggesting that these may be important sites associated with the host immune response. In addition, sites 513 in HA and 339 in NA were positively selected in the 2023 dataset, and were never detected in a positive selection analysis in previous studies [28,33,34]. Acquisition/loss of glycosylation at position 513 in HA was observed in a few strains isolated from India in 2018 and called for further investigation [35]. Analysis of the amino acid sequences of 25 in NA isolated from China during the 2023 season showed that residue 339 had changed in all viruses compared to vaccine strain A/Wisconsin/67/2022, suggesting stronger selection pressure at this site [36].

The results of this study show a gradual adaptation of the 2009 pandemic A(H1N1) influenza virus as a seasonal strain. However, the virus continues to circulate in the population, and its evolutionary rates should be continuously monitored to maintain its virological surveillance. 

## 5. Conclusions

We report here on the evolutionary dynamics and genetic diversity of the structural glycoproteins HA and NA of A(H1N1) pdm09 viruses in Italy since the 2009 pandemic, which has established itself as a seasonal strain with gradual mutation. During the 2009 pandemic, pdm09 viruses of clade 6B.1 dominated, while the 2023 season was characterized by the dominance of new viruses of the clade 6B.1A.5a.2a and its descendant 6B.1A.5a.2a.1. In light of the evolutionary dynamics observed in the main genes of the influenza virus A(H1N1) pdm09 in Italy, it is imperative to consider the broader implications for viral adaptation and its impact on human populations. The simulation results indicate a notable shift in the distribution of sites under selective pressure over time. The observed increase in sites undergoing negative selection within the HA gene suggests a potential evolutionary trend towards a less virulent viral form. This phenomenon could be attributed to selective pressures favoring variants that are less detrimental to host survival, potentially indicating the development of adaptive strategies to evade host immune responses. Conversely, the decrease in sites under positive selection in both HA and NA genes may signify an equilibrium between the virus and its host. This equilibrium could reflect the emergence of strategies to evade host immune surveillance or an effective immune response targeting previously positively selected sites. Furthermore, the consistent ratio of synonymous to non-synonymous mutations, with synonymous mutations being more frequent, suggests a relatively stable protein sequence with conservative selection pressure. This equilibrium may be maintained by a dynamic interplay between various evolutionary factors, including negative selection, genetic drift, and recombination. It is crucial to recognize that the observed evolutionary patterns may be influenced by random selection events or genetic bottleneck occurrences, which could further shape the distribution of sites under selective pressure over time.

Overall, our observations provide important insights into the evolution of the virus and can provide strategies for disease control, prevention, and the development of vaccines, therapies, and, in general, devising effective strategies for mitigating the impact of viral evolution on human health.

## Figures and Tables

**Table 1:** Site under positive selective pressure. α: mean posterior synonymous substitution rate at a site; β: mean posterior non-synonymous substitution rate at a site; Prob [α > β]: posterior probability of negative selection at a site. Prob [α < β]: posterior probability of positive selection at a site; BF [α < β]: Empirical Bayes Factor for positive selection at a site; β-α: the difference between the non-synonymous and synonymous substitutions indicating the selective pressure on protein-coding sequences.

	Site	nt Position	α	β	Prob [α > β]	Prob [α < β]	BF [α < β]	β-α
HA_2009	177	529–531	0.895	6.484	0.025	0.945	24.681	5.589
	41	121–123	1.077	9.591	0.030	0.942	23.428	8.514
HA_2023	123	367–369	0.880	6.570	0.049	0.918	19.964	5.690
	22	64–66	0.889	5.870	0.058	0.906	17.231	4.981
	513	1537–1539	0.926	5.960	0.059	0.903	16.787	5.034
NA_2009	286	856–858	0.942	10.977	0.010	0.970	47.275	10.035
	248	742–744	0.957	10.843	0.011	0.969	45.162	9.886
	455	1363–1365	5.037	28.782	0.048	0.906	14.059	23.745
NA_2023	339	1015–1017	1.021	13.242	0.049	0.925	18.000	12.221

## Data Availability

The data presented in this study are available on request from the corresponding author.

## Supplementary Materials

The following supporting information can be downloaded at: https://www.mdpi.com/article/10.3390/pathogens13040334/s1, Figure S1: Extended form of the time-scaled phylogenetic tree shown in Figure 1. The tree includes all of the Italian A(H1N1) pdm09 HA sequences sampled between 2009 and 2023. All nodes are highly supported (posterior probabilities > 0.95). The scale below is expressed in years before 2023; Figure S2: Extended form of the phylogenetic reconstruction of Italian isolates of influenza A H1N1pdm09 HA gene sequences circulating in Italy in 2009; Figure S3: Extended form of the phylogenetic reconstruction of Italian isolates of influenza A H1N1pdm09 HA gene sequences circulating in Italy in 2023; Figure S4: Extended form of the phylogenetic reconstruction of Italian isolates of influenza A H1N1pdm09 NA gene sequences circulating in Italy in 2009; Figure S5: Extended form of the phylogenetic reconstruction of Italian isolates of influenza A H1N1pdm09 NA gene sequences circulating in Italy in 2023.
