# Supplementary figures and images for: Phylodynamic and Evolution of the Hemagglutinin (HA) and Neuraminidase (NA) Genes of Influenza A(H1N1) pdm09 Viruses Circulating in the 2009 and 2023 Seasons in Italy
Source: Pathogens. 2024 Apr 17;13(4):334. doi: 10.3390/pathogens13040334 (PMC11054071; doi:10.3390/pathogens13040334)

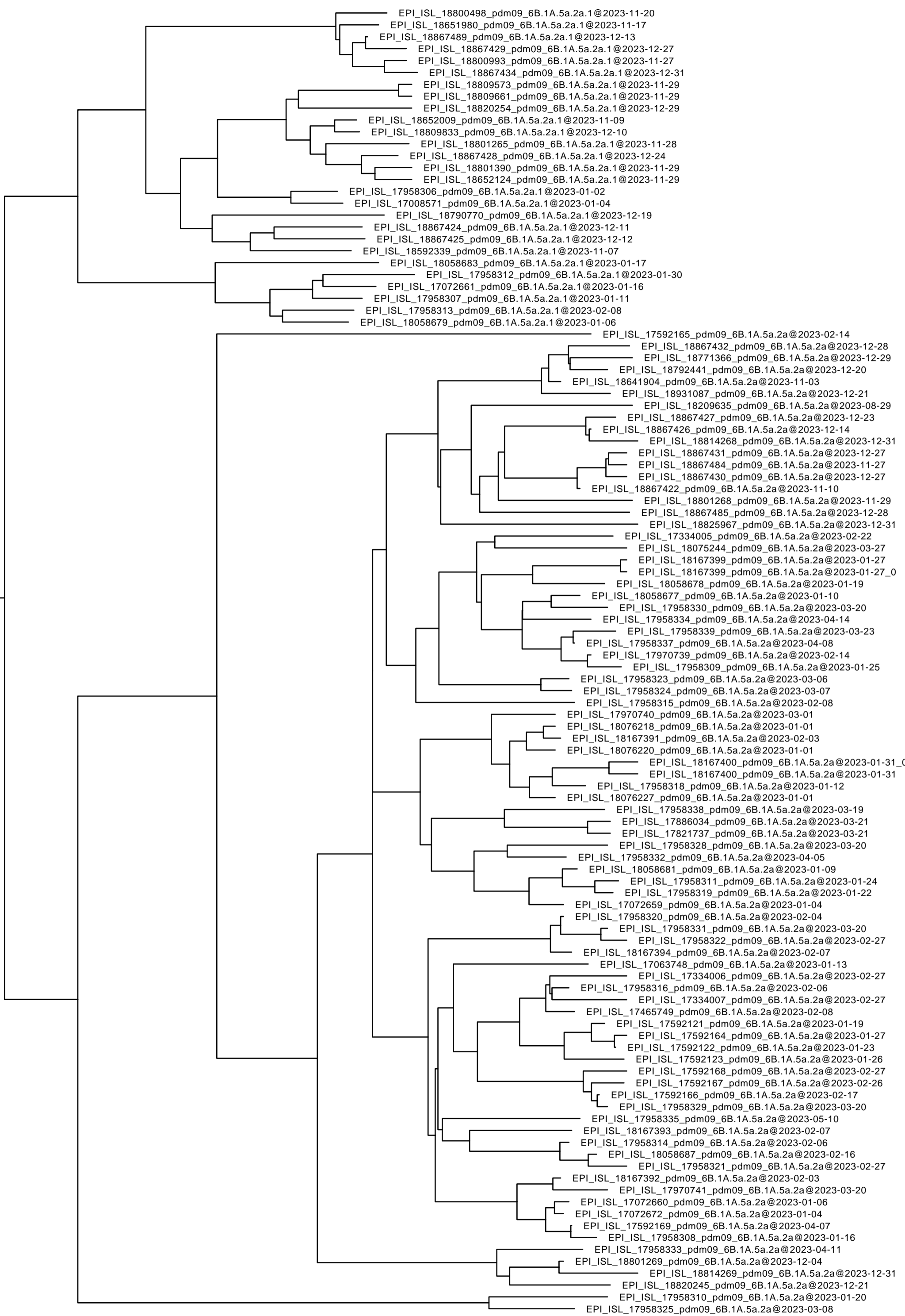

0.08

Supplement: Supplementary file 1 [file pathogens-13-00334-s001.zip › Suplementary_file/Figure_S3.pdf]

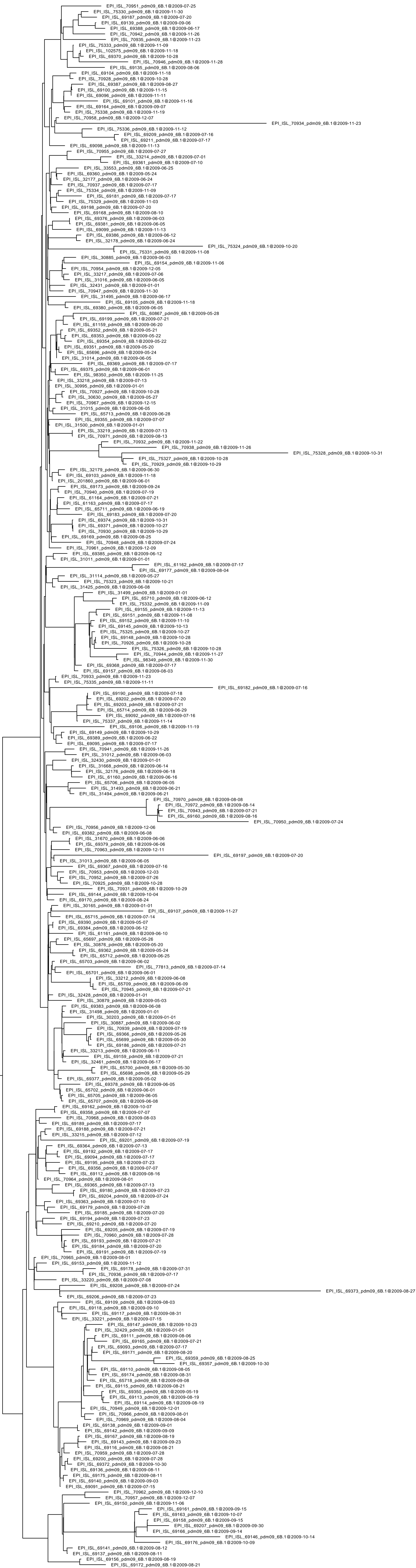

Supplement: Supplementary file 1 [file pathogens-13-00334-s001.zip › Suplementary_file/Figure_S2.pdf]

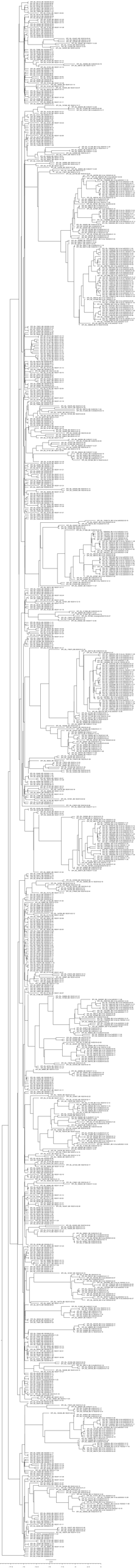

Supplement: Supplementary file 1 [file pathogens-13-00334-s001.zip › Suplementary_file/Figure_S1.pdf]

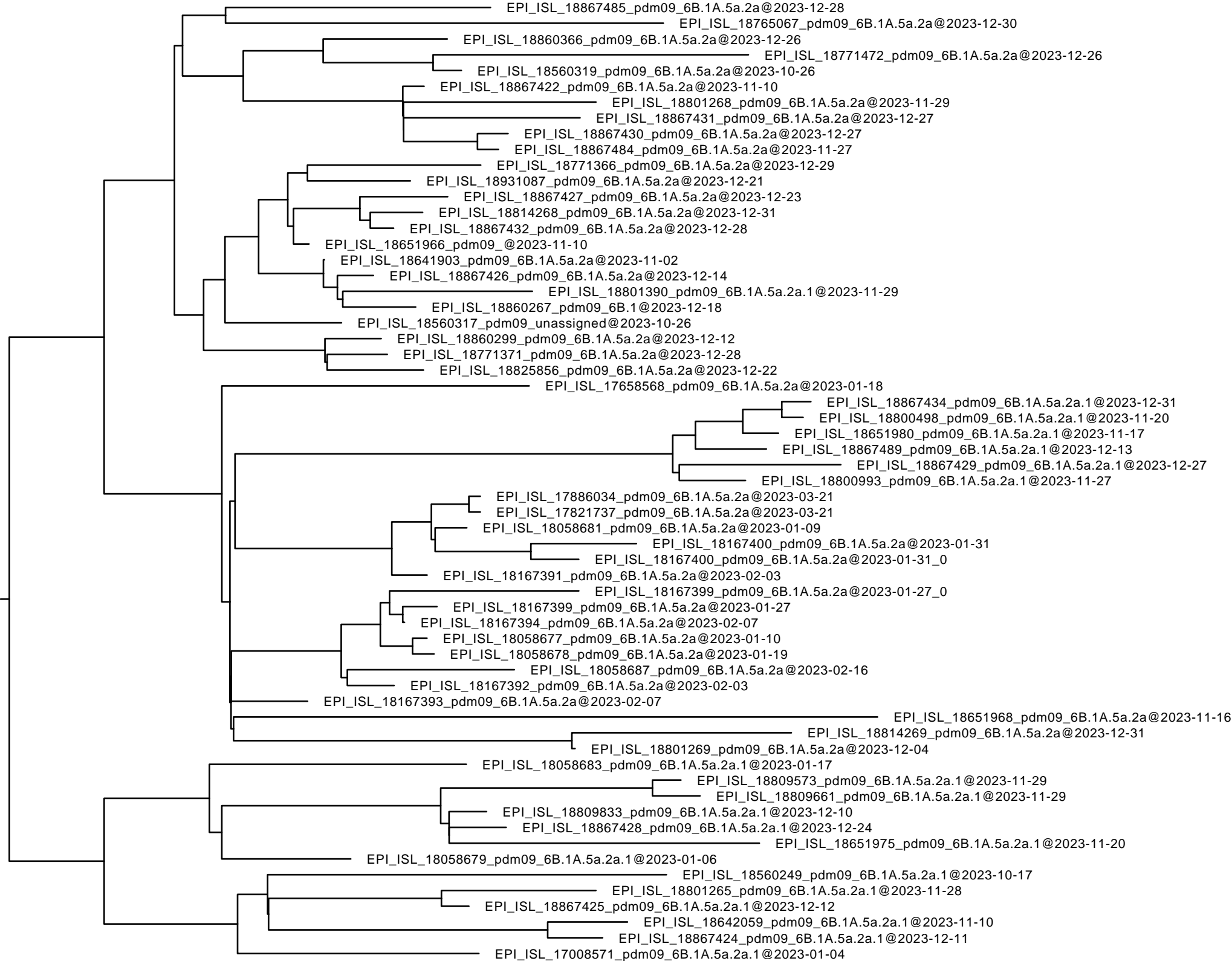

7.0E-4

Supplement: Supplementary file 1 [file pathogens-13-00334-s001.zip › Suplementary_file/Figure_S5.pdf]

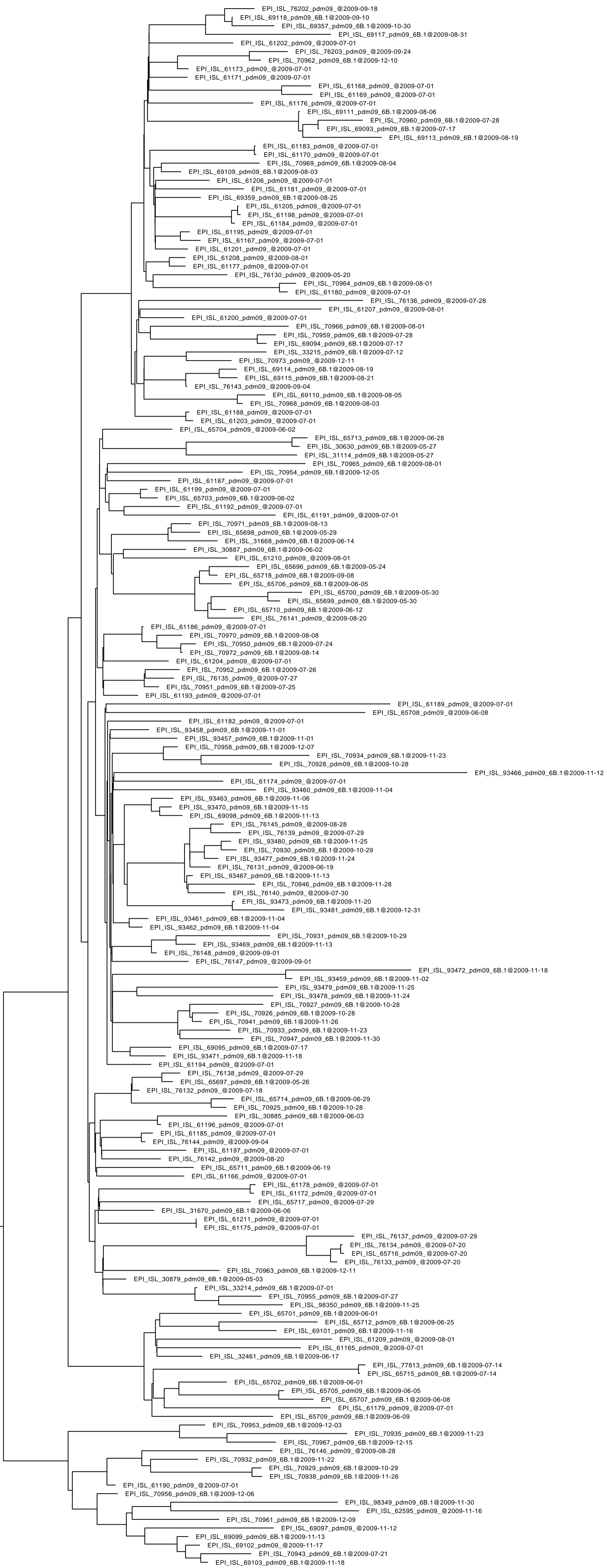

4.0E-4

Supplement: Supplementary file 1 [file pathogens-13-00334-s001.zip › Suplementary_file/Figure_S4.pdf]
